# Machine Learning-Based Radiomics Analysis for Identifying KRAS Mutations in Non-Small-Cell Lung Cancer from CT Images: Challenges, Insights and Implications

**DOI:** 10.3390/life15010083

**Published:** 2025-01-11

**Authors:** Mirjam Schöneck, Nicolas Rehbach, Lars Lotter-Becker, Thorsten Persigehl, Simon Lennartz, Liliana Lourenco Caldeira

**Affiliations:** Institute for Diagnostic and Interventional Radiology, Faculty of Medicine and University Hospital Cologne, University of Cologne, 50937 Cologne, Germany

**Keywords:** radiomics, NSCLC, Kirsten Rat Sarcoma viral oncogene homolog (KRAS), machine learning, transfer learning

## Abstract

Kirsten Rat Sarcoma viral oncogene homolog (KRAS) is a frequently occurring mutation in non-small-cell lung cancer (NSCLC) and influences cancer treatment and disease progression. In this study, a machine learning (ML) pipeline was applied to radiomic features extracted from public and internal CT images to identify KRAS mutations in NSCLC patients. Both datasets were analyzed using parametric (*t* test) and non-parametric statistical tests (Mann–Whitney U test) and dimensionality reduction techniques. Afterwards, the proposed ML pipeline was applied to both datasets using a five-fold cross-validation on the training set (70/30 train/test split) before being validated on the other dataset. The results show that the radiomic features are significantly different (Mann–Whitney U test; *p* < 0.05) between the two datasets, despite the use of identical feature extraction methods. Model transferability is therefore difficult to achieve, which became evident during external testing (F1 score = 0.41). Oversampling, undersampling, clustering and harmonization techniques were applied to balance and harmonize the datasets, but did not improve the classification of KRAS mutation presence. In general, due to only a single moderate result (highest test F1 score = 0.67), the accuracy of KRAS prediction is not sufficient for clinical application. In future work, the complexity of KRAS mutation might be addressed by taking submutations into consideration. Larger multicentric datasets with balanced tumor stages, including multi-scanner datasets, seem to be necessary for building robust predictive models.

## 1. Introduction

The global cancer statistics for 2022 estimate a cumulative risk of 20.1% for developing cancer before the age of 75. Lung cancer is the most common and fatal cancer among men, and the second most common in women after breast cancer, accounting for 12.4% of all cancer cases across both sexes [1]. It can be distinguished into small-cell (SCLC) and non-small-cell lung cancer (NSCLC), with NSCLC being the focus of this study [2]. The most common subtypes of NSCLC are squamous cell carcinoma, large cell carcinoma, and adenocarcinoma [3]. Genetic mutations can accompany NSCLC, with Epidermal Growth Factor Receptor (EGFR) and Kirsten Rat Sarcoma viral oncogene homolog (KRAS) being two of the most frequently occurring ones. Since such mutations impact the course of the disease and patient survival, early knowledge can enhance the physician’s treatment decisions, making early detection crucial to combat disease progression [4].

Our work focuses on the mutation of KRAS which, despite medical advances, is difficult to inhibit [5]. Typically, KRAS is identified using tissue samples, e.g., through CT-guided transthoracic biopsy. Due to the potential complications of this procedure (such as pneumothorax and pulmonary hemorrhage, which may both be life-threatening [6,7]), non-invasive biomarkers are of high clinical interest. As a surrogate, plasma samples have been investigated, which showcased high specificity and moderate sensitivity (0.94 and 0.71, respectively) in capturing KRAS mutations based on a review by Cai et al. [8]. Radiomics have also been explored as an emerging field of research to predict the molecular or genetic characteristics of tumors, potentially complementing non-invasive biomarkers.

Radiomics describes a method to extract quantitative information from diagnostic images. It enables the analysis of lesions, organs, or other structures of interest, in terms of quantitative features such as size, volume, heterogeneity or density, allowing for an analysis of complex patterns [9]. Additionally, radiomic features are mineable, enabling the creation of databases that can be statistically analyzed or used for machine learning (ML) algorithms in prediction tasks [10]. The subdisciple focusing on combining these features with genomic information is called radiogenomics [11]. As the field continues to evolve, groups such as the image biomarker standardisation initiative (IBSI) aim to standardize radiomic features, allowing for study reproducibility and data consistency [12]. Typical radiomic features include intensity, shape, and texture-based information, which can be furthermore expanded using image preprocessing techniques such as wavelet filtering [10].

As large amounts of annotated high quality medical data are often not available and ML performance typically benefits from more observations, model transferability is a relevant subject in medical research. This is especially prevalent when medical institutions would like to perform ML-based analyses on comparably smaller patient cohorts, due to a rare disease being the focus of the study or due to a missing local specialization in the use case. Since ML models are trained on a specific dataset from one or more clinical institutions, the question arises whether the models can perform equally well on novel data from another institution without requiring any retraining on local data. One aspect to consider is that the data may have an inherent bias, e.g., caused by different image acquisition or reconstruction protocols or hardware.

In this study, we investigate the radiomic signature of KRAS mutations and the transferability of classification models in NSCLC by taking a publicly available NSCLC dataset [13] from The Cancer Imaging Archive (TCIA) [14] and a local dataset into account for model training and testing. First, dataset characteristics are compared in terms of radiomic features; afterwards, classification model transferability is tested in both directions. The pipeline for building a ML model to classify KRAS mutation in NSCLC includes the approaches of Moreno et al. [15], Le et al. [16], Pinheiro et al. [17], and Prencipe et al. [18].

## 2. Related Work

Several studies have explored the application of machine learning in lung cancer for virtual biopsy or clinical outcome prediction [19]. Works aiming to classify EGFR and KRAS mutation status in NSCLC utilizing ML-based radiomics or deep learning approaches are present in literature, including the work of Liu et al. [20], Dong et al. [21], Koyasu et al. [22], Morgado et al. [23], and others. Throughout this paper, the focus is on studies using ML that report KRAS classification results based on the TCIA dataset NSCLC Radiogenomics  [13] and their performance.

Shiri et al. [24] used CT images of 150 patients from the TCIA dataset to train ML classifiers to predict EGFR and KRAS mutations status. This resulted in an Area Under the Curve (AUC) of 0.83 for KRAS classification using CT images. Le et al. [16] extracted radiomics and applied several ML algorithms and feature selection methods based on 161 patients. Combining XGBoost with a genetic algorithm for feature selection resulted in the most favorable performance, showcasing an AUC of 0.81. Moreno et al. [15] used ML algorithms and Convolutional Neural Networks (CNNs) as classifiers on a dataset of 83 patients. They furthermore introduced ensemble algorithms and voting schemes into their pipeline, presenting ML-based AUC values of 0.65 using a Support Vector Machine (SVM) and 0.71 using the ensemble voting approach. Pinheiro et al. [17] used ML and dimensionality reduction techniques to analyze and classify EGFR and KRAS mutations using a subset of 114 patients. Besides radiomics, they utilized semantic information present in the dataset for classification as well. Contrary to their EGFR results (an AUC of 0.75 using hybrid semantic features), the complexity of the KRAS mutation does not allow for visual or ML based distinctions between the two classes, resulting in a mean AUC of 0.51 over 100 data splits using XGBoost. Prencipe et al. [18] focused solely on a subset of adenocarcinoma patients and combined the TCIA dataset with an internal cohort, totaling 115 patients. Their results showcase promising transfer learning and dataset merging results, with an AUC of 0.82.

## 3. Materials and Methods

### 3.1. Datasets

#### 3.1.1. Internal Dataset

The institutional review board approved this study and waived the necessity to obtain informed consent due to the retrospective study character. By means of a systematic query of the picture archiving and communication system (PACS), patients meeting the following criteria were selected: oncologic patients of 18 years age or older, with a histopathologically confirmed NSCLC diagnosis, and venous phase chest CT between July 2010 and July 2019. One lesion was included per patient.

In total, the internal dataset comprises 212 conventional CT images acquired on different CT scanners, along with molecular information for each lesion (mutation statuses of KRAS, EGFR, MET protooncogene, Anaplastic lymphoma kinase (ALK), ROS protooncogene and Fibroblast Growth Factor Receptor (FGFR)). The image datasets were transferred to a proprietary software (Philips IntelliSpace Discovery Version 3.0, Best, The Netherlands) and annotated by a medical doctorate student (L.L.-B.) after use-case-specific training and under the supervision of a board-certified radiologist with more than 5 years experience (S.L.) in chest imaging. A consensus read with a board-certified radiologist (T.P.) (>15 years experience) was performed for critical cases. During the annotation process, special attention was given to the tumor outlines to avoid inclusion of healthy lung parenchyma or air.

#### 3.1.2. TCIA Dataset

The publicly available dataset NSCLC Radiogenomics from TCIA was published by Bakr et al. [13] and is used in several studies to analyze EGFR and KRAS mutations. This dataset comprises (among other clinical, semantic and image-based information) conventional CT images, tumor annotations, and information on the gene mutation statuses of EGFR, KRAS, and ALK, collected retrospectively from 2008–2012. From 211 available subjects, 144 provide tumor segmentations, which were first generated using an automatic segmentation algorithm before being verified and if necessary edited by two radiologists using ePAD [25]. Being a retrospective study, the scanners and scanning protocols deviate, resulting in differing slice thickness and X-ray tube currents. Further details describing the data are given in the paper of Bakr et al [13].

### 3.2. Data Preprocessing

**Figure 1:**
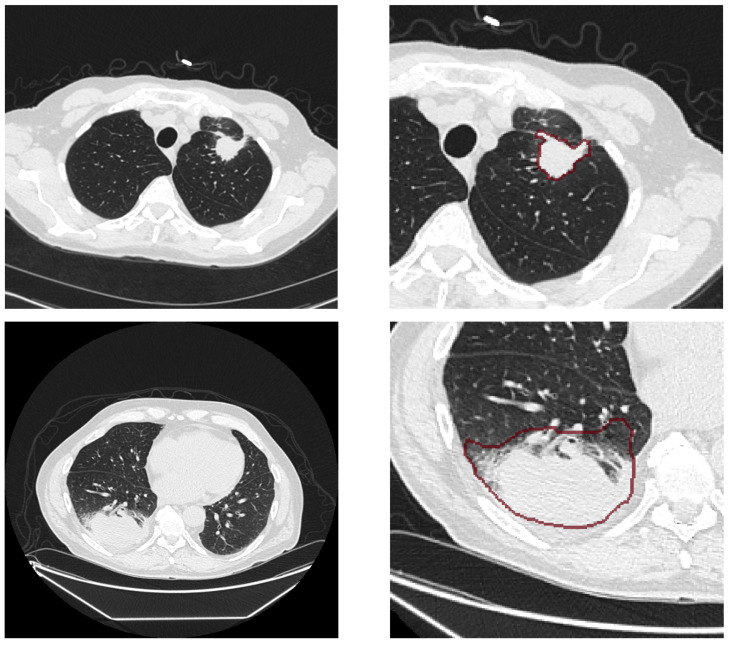
A visualization of CT images (**left**) and their corresponding segmentation (**right**, indicated by red line) from the TCIA dataset, showcasing the differences in segmentation precision and the inclusion of air or lung parenchyma in R01-075 (**top**) and R01-069 (**bottom**).

While the software IntelliSpace Discovery allowed for the direct NIfTI export of the locally collected imaging data, the TCIA dataset was converted from DICOM to NIfTI using the Python library dicom2nifti [26]. The segmentations were transformed using pydicom-seg [27] and dcmqi [28]. Following the conversion, the congruence of images and segmentations was visually ensured using a local library, as visualized in Figure 1. Differences in the slice thickness, incorrect image orientation, and other mismatches in the image-segmentation pairs were corrected, resulting in a dataset of 143 patients, of whom 114 included information about the KRAS mutation status.

### 3.3. Radiomic Feature Extraction and Analysis

Radiomic features were extracted using PyRadiomics [29] (version 3.1.0, python 3.9.0) with standard extraction parameters for CT images and specifically selected extraction parameters optimized for the internal dataset. The optimized PyRadiomics extraction parameters included: resampled voxel size: 1 × 1 × 1 mm³ using b-spline interpolation (same as default); bin size: 20 (default: 25); resegmentation range: [−550,700] (default: no resegmentation); voxel array shift: 1024 (default: 1000); features: firstorder, shape, glcm, gldm, glrlm, glszm, ngtdm (feature class names: glcm: gray level co-occurrence matrix, gldm: gray level dependence matrix, glrlm: gray level run length matrix, glszm: gray level size zone matrix, ngtdm: neighbouring gray tone difference matrix) (same as default); image types: original. These optimized parameters were chosen based on a thorough visual analysis of the grey value distributions inside the segmented regions, such that grey value histograms yielded between 30 and 130 non-empty bins (as recommended in PyRadiomics), and the resegmentation range only cut off outlier voxels. As this study focuses on radiomics only, additional information such as subject age, histology, or smoking status present in the TCIA dataset was omitted.

To further validate data integrity and analyze differences in both datasets, visual and statistical analyses were conducted. Aside from portraying differences in the data, this ensures that the TCIA segmentations are not corrupted by adjusting the resegmentation range, which was set in order to exclude air voxels from the segmentations. First, the extracted image properties and shapes were analyzed using descriptive summaries and visualizations to discover differences in the datasets. In a second step, the Mann–Whitney U test was applied to observe if a significant difference in the shape features of both datasets exists. This approach furthermore allows us to detect outliers in the datasets.

Finally, to understand the differences between the datasets on a global level, Principal Component Analysis (PCA) and t-Distributed Stochastic Neighbor Embedding (t-SNE) were applied. In PCA, dimensions are reduced to a chosen number of principle components utilizing linear combinations of the present dataset [30]. Following this, t-SNE is utilized, a method to reduce and visualize high dimensional spaces while preserving the local structure of the underlying data [31]. Van der Maaten and Hinton refer to dimensionality reduction as the aim to project as much of the significant information and structure into a low dimensional space [31]. Dimensionality reduction can therefore reveal insightful information. However, it is important to recognize that the process can also lead to information loss due to the data compression affecting model performance.

### 3.4. Data Splits

In total, seven variations of the two datasets have been created for further analyses; two based on the TCIA dataset, two based on internal data, and three that consider a combination of both datasets. The variation referred to as “TCIA” in the analysis is based on the 114 relevant patients of the extracted TCIA data and consists of original radiomic feature extraction. To validate the ML pipeline and the reproducibility of the study, a dataset was included from Moreno et al. [15], who published their radiomic data based on the TCIA dataset. Their data was used originally, aside from filling in nan values for two rows in *GLSZM_Small zone emphasis* and *GLSZM_Small zone high grey level emphasis* with the columns respective means, which will be referred to as “Moreno et al.” in the analysis. Based on the internal dataset, two variations were created: a dataset consisting of the extracted radiomic features of 212 subjects referred to as “Internal”, and a randomly undersampled balanced subset “Internal_balanced_”. As both of the extracted datasets are unbalanced, with 24% (TCIA) and 27% (internal) of the subjects having KRAS mutations, a balanced dataset was created from the internal dataset, consisting of 115 subjects with evenly distributed mutation statuses. As a result of the PCA, two subsets of the combined internal and public dataset were investigated further. These subsets were computationally defined by clustering the t-SNE visualization using the density-based spatial clustering of applications with noise (DBSCAN) [32] and are therefore referred to as “Cluster 0” and “Cluster 1”, including 161 and 165 subjects respectively. DBSCAN was chosen as the clustering method because its density-based approach effectively differentiates between the two clusters in a visually ideal manner. Besides splitting the data, the extracted features from the combined dataset were grouped by their data source and then harmonized using ComBat [33], implemented by Fortin and Foran [34,35,36], to account for batch effects. This dataset will be referred to as “Harmonized” and contains all 326 patients. All of the datasets have been split in a 70/30 train–test manner, given in Table 1, as the minority class would otherwise be strongly underrepresented and would not allow for robust testing.

### 3.5. Feature Selection

Similar to the work of Moreno et al. [15] and Le et al. [16], eight feature selection methods were applied by specifying the desired number of features to be selected. After experimenting with different numbers of features, 3, 5, 10, and 15 were chosen in the final analysis to assess a broad range of features while minimizing the potential of overfitting, as the datasets comprise between 83 and 212 patients. The feature selection algorithms chosen were KBest, Recursive Feature Elimination (RFE), Pearson correlation, Spearman correlation, Mann–Whitney U (MW) implemented in scikit-learn  [37], Minimum Redundancy Maximum Relevance (MRMR) by Mazzanti [38], Genetic Algorithm (GA) by Gómez [39], and Relief by Urbanowicz et al. [40]. Prior to applying the algorithms, highly correlated features were removed randomly using Pearson’s correlation coefficient (r>0.9) to mitigate feature redundancies in the classification task.

### 3.6. Data Augmentation

The Synthetic Minority Over-sampling Technique (SMOTE) [41] is a technique to generate synthetic samples of the minority class in an unbalanced dataset, therefore allowing for balanced training of both classes. As the datasets were unbalanced and KRAS mutations were underrepresented, SMOTE (implemented by Lemaître et al. [42]) was applied for each cross-validation fold individually to mitigate information leakage before fitting the classification algorithms. Blagus and Lusa [43] showed that SMOTE is more beneficial on low dimensional data for most classifiers. Therefore, SMOTE was applied after feature selection. While SMOTE was applied to all training sets but the balanced one, the test data always remained solely standardized.

### 3.7. Machine Learning Pipeline

**Figure 2:**
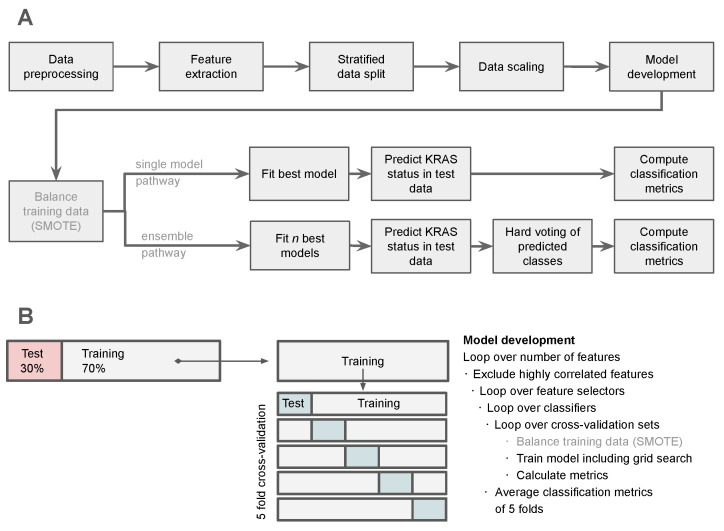
A visualization of the applied machine learning pipeline including the two evaluation pathways, using either a single classification model or an ensemble classifier (**A**). For model development, the training data is split into a five-fold cross-validation, where multiple numbers of features, feature selectors, and classifiers are tested and optimized using grid search (**B**). SMOTE is only applied to unbalanced datasets.

Following the generation of the different dataset variations and the decision on the applied feature selection methods, a ML pipeline was created as visualized in Figure 2. The ML pipeline was constructed as follows:

First, the training and test sets were standardized using a z-transformation fitted on the training set. After selecting 3, 5, 10, or 15 features, respectively, the training data was further split into a stratified five-fold cross-validation loop. For each fold, all possible feature selector and classifier combinations were tested. Where applicable, meaning in all datasets but the balanced one, the data augmentation with SMOTE was applied right after feature selection and before the fitting of the classifier. To find the best parameter combination, a grid search using the function GridSearchCV() from the package scikit-learn [37] was applied for each fold, maximizing for the F1 score. The F1 score was chosen as the main metric, since it balances precision and sensitivity and is therefore well suited to analyze imbalanced class problems.

The F1 score is defined as

$$F1\;\mathrm{Score}=2\times \frac{\mathrm{Precision}\times \mathrm{Recall}}{\mathrm{Precision}+\mathrm{Recall}},\;\mathrm{where}\;\mathrm{Precision}=\frac{\mathrm{TP}}{\mathrm{TP}+\mathrm{FP}}\;\mathrm{and}\;\mathrm{Recall}=\frac{\mathrm{TP}}{\mathrm{TP}+\mathrm{FN}}$$

with TP: true positives, FP: false positives, FN: false negatives.

The classification algorithms included K-Nearest Neighbors (KNN), Random Forest (RF), Logistic Regression (LR), Extreme Gradient Boosting (XGBoost) and Neural Networks (NN) implemented by scikit-learn [37].

To assess model performance on the training data, metrics were first computed in terms of the model’s accuracy, Receiver Operating Characteristic AUC, F1, sensitivity, and specificity based on each model’s predictions within the five cross-validation folds, and then averaged among the five folds. The best-performing model was assessed based on the highest achieved F1 score on a fold during cross-validation. For each combination of a classifier and feature selection algorithm, the trained model and its parameters, used features, and performance were saved in a table. To evaluate the performance of the trained models on unseen data, the best-performing models within the cross-validation were refitted on the whole training set with optional SMOTE before testing (referred to as single model pathway in Figure 2).

As Moreno et al. [15] showcased improving results using an ensemble approach, a similar method was implemented for the test set (referred to as ensemble pathway in Figure 2). Based on the best-performing models, an ensemble was created with an agreement threshold parameter (e.g., 60%). If the agreement threshold was reached by the majority of models predicting the minority class, this class was assigned to that subject. The models were each refitted on the whole training dataset utilizing the best-performing parameter configuration from the cross-validation and predicted the mutation status of a subject in the test set in a hard voting manner.

## 4. Results

### 4.1. Dataset Comparison and Statistical Analysis

#### 4.1.1. Feature Extraction Parameters

Prior to using extracted features in a ML pipeline, it was ensured that the choice of feature extraction parameters did not result in substantial differences. Therefore, the mask information was compared for radiomic features extracted with default parameters and features extracted with the optimized internal parameters. The feature *original_shape_VoxelVolume* showed an overall mean volume decrease of 0.8% with a standard deviation of 1.7% in the dataset when being extracted with the optimized parameters. Also, when comparing the *original_shape_Sphericity*, a mean increase of 1.1% was observed with a standard deviation of 1.7%. Furthermore, the differences in the number of voxels and sphericity was tested for statistical differences. Due to the non-normal distribution of the feature *original_shape_VoxelVolume*, the Mann–Whitney U test was applied, which revealed no significant differences (p=0.91) among the extraction types. For the feature *original_shape_Sphericity*, a *t* test was applied due to normal distribution of the data, resulting in no significant difference as well (p=0.66). These results indicate that there is no significant difference in the datasets with respect to the extraction method. Thus, to allow for ideal model transferability, the extraction parameters used for the internal dataset were also applied when extracting radiomics for the TCIA dataset.

#### 4.1.2. Mask Resegmentation

**Figure 3:**
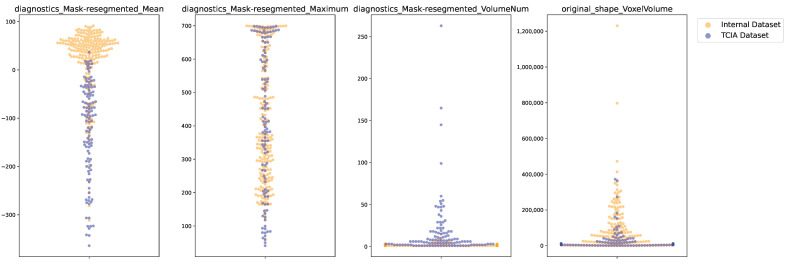
A visualization of swarmplots portraying the differences in both datasets for the region of interests, including its mean and maximum intensity, number of connected volumes, and total volume (from left to right).

In a second step, both datasets were compared in terms of their resegmented image properties and shape features. As the assumption for normal distribution was again violated for each of the features, the Mann–Whitney U test was applied to analyze if a statistical difference was present for the diagnostic features *diagnostics_Mask-resegmented_Mean*, *diagnostics_Mask-resegmented_Minimum*, *diagnostics_Mask-resegmented_Maximum*, *diagnostics_Mask-resegmented_VolumeNum*. Besides the *diagnostic_Mask-resegmented_Maximum* (p=0.68), all features showed a significant difference (p<0.05), which is also visually observable in Figure 3.

#### 4.1.3. Principal Component Analysis

**Figure 4:**
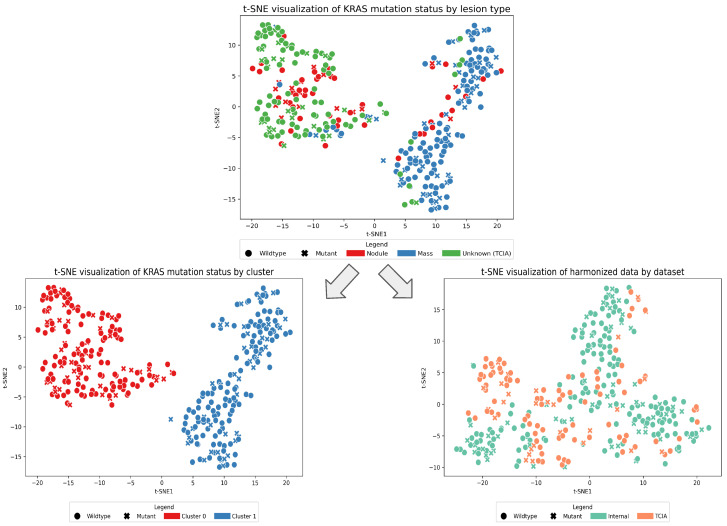
Three scatterplots showcasing the global data distribution reduced using PCA and t-SNE. The t-SNE plot in the top row visualizes the data distribution divided by mutation and lesion type, excluding any shape features. After discovering the clusters existent in the underlying data, a clustered dataset (bottom left) and harmonized dataset (bottom right) were created.

To analyze the global structure, the datasets were merged resulting in 105 radiomic features of 326 cases. These features were reduced to 10 principal components using PCA, followed by dimensionality reduction with t-SNE. The results revealed a distinction into two clusters, with the TCIA dataset being mainly clustered on the left side (t-SNE1 <0) mixed with observations of the internal data. However, a larger fraction of the internal data was spread across the right side (t-SNE1 >0), varying strongly on the y-axis. A differentiation between the KRAS mutation status cannot be observed, which would be the outcome variable of interest. Further analysis was conducted to investigate why the two observed clusters were present. As information about the lesion type was present for the internal data, a visual analysis was conducted using the same methodology. This revealed that the majority of the internal nodules cluster with the TCIA data, while masses predominantly form a cluster with higher t-SNE1 values. As shape features would be the most likely to indicate differences in nodules and masses, the 14 extracted shape features have been excluded to avoid a distinction based on lesion size. Nonetheless, the pattern remained as visualized in Figure 4. This finding motivated further research and led to the construction of three datasets. First, a clustering-based differentiation yielded two subsets, so that expert models were trained for each cluster. Second, we attempted to harmonize the extracted features using ComBat by considering batch effects from different data sources.

### 4.2. KRAS Mutation Classification

#### 4.2.1. Training Performance

The training’s cross-validation results presented in Table 2 showcase mean accuracies ranging from 0.45±0.11 to 0.70±0.15. Similar results were present for the mean AUC, with values from 0.43±0.05 to 0.69±0.15 with the best-performing models in this metric being two LR models trained on the dataset by Moreno et al. using three features selected by the KBest and Pearson correlation algorithms. The models not trained on the balanced dataset showed comparably lower F1 scores ranging from 0.34±0.07 to 0.54±0.11 with standard deviations up to 0.22, depending on the dataset. A difference can be observed for the model trained on the balanced dataset, with each model resulting in a mean F1 of 0.68±0.02 (0.68±0.04, respectively) due to a one-sided classification. Besides the models trained on the balanced dataset, many models yielded high variability in all of the metrics, indicating fluctuating performance depending on the cross-validation data split. It is noteworthy that these fluctuations are more extreme for the models trained on the TCIA dataset (e.g., with standard deviations of up to 0.33 in the mean sensitivity).

Also, the datasets generated by clustering did not lead to improved performance, suggesting that this method did not improve the models’ capabilities to differentiate KRAS mutants from wildtypes compared to using the full dataset. While the use of ComBat as a harmonization technique resulted in more stable training, indicated by reduced variability in the metrics, it did not result in a better overall training performance.

#### 4.2.2. Test Performance

The best-performing models of the cross-validation were subsequently applied to the test datasets using single model prediction and the ensemble method. The results are given in Table 3.

Similar to the training results, none of the best performing models from the training led to satisfactory results during prediction on the test datasets regarding the F1 score. Although SMOTE and undersampling were applied to mitigate the bias in the underlying data during training, none of the models learned to robustly differentiate between the classes. This is observable by comparably high sensitivity or specificity in some models, but no co-occurrence of both, which is reflected by the generally low F1 scores. This is especially noticeable when training and test datasets differed. The best-performing model combination in terms of F1 score was found when training and testing on the balanced internal dataset. An ensemble of five models achieved a F1 score of 0.67 and an AUC of 0.53. Nonetheless, the specificity of 0.06 again portrays the clear bias, meaning that the ensemble tended to predominantly classify lesions as mutants. In this regard, the model trained and tested on the unbalanced internal dataset showed superior performance, with a sensitivity of 0.72 and a specificity of 0.52.

Noteworthy, the KNN model trained on the dataset of Moreno et al. utilizing the MRMR algorithm for feature selection yielded the highest accuracy of this study (0.68), a sensitivity of 0.67, and a specificity of 0.68. This indicates that the classifier is less biased towards one class in comparison to its counterparts. The XGBoost model trained on the TCIA dataset attained the highest AUC (0.62); however, it failed to deliver satisfactory results in terms of its F1, sensitivity, or specificity scores. Similarly, the models trained on the clustering based datasets did not achieve any acceptable results, portraying the comparably lowest test F1 scores of the study. Furthermore, the harmonization of the datasets did not aid classification abilities in a test setting.

In terms of transferability, the models trained on the TCIA dataset and applied to internal data (and vice versa) present low classification abilities, with the best TCIA based model demonstrating an accuracy of 0.60, AUC of 0.47 and F1 of 0.21 on the internal data. Applying the best-performing model based on the internal dataset to the TCIA dataset results in an accuracy of 0.46, an AUC of 0.55, and an F1 of 0.41. Also, training on the balanced internal dataset only results in an accuracy of 0.25, an AUC of 0.44, and an F1 of 0.39. The utilization of the model ensemble did not improve the results, indicating that the individual models differentiate between the two classes equally well. This is observable for the models trained on the dataset of Moreno et al., where the comparably good results of the KNN classifier are suppressed through the majority voting.

### 4.3. Computational Costs

The aforementioned segmentation of images, the extraction of radiomic features, and the deployment of the ML pipeline has been run on standard workstations from our radiology department and does not require any specific high-performance computation resources. In case image segmentation would be carried out semi-automatically, e.g., by making use of an artificial intelligence (AI)-assisted presegmentation, GPU resources might be required depending on the software package. All other processing steps run on CPU resources, making this pipeline applicable in a clinical environment.

## 5. Discussion

### 5.1. Dataset Comparison

The two investigated datasets, one internally acquired dataset and a public one from TCIA, were found to be dissimilar and statistically different from each other. When investigating basic image parameters, based on extracted features from the class *diagnostics_Mask-resegmented_*, the mean Hounsfield units (HU) of the masked areas were higher in the internal dataset compared to the public dataset. This could originate from narrower segmentations around the tumor, meaning less inclusion of air or lung parenchyma, the unintended inclusion of denser areas (such as bone), or it could relate to denser lesions themselves. Additionally, a discrepancy was observed for the feature *diagnostics_Mask-resegmented_VolumeNum*, revealing that the segmentations of the internal dataset largely consist of single or very few coherent segmentations, while the TCIA segmentations can be fragmented to a higher degree. The feature extraction from multiple discontiguous regions of interest, which are considered to form one single lesion, can lead to less meaningful features, which is especially relevant for shape and texture features.

#### 5.1.1. Feature Extraction Parameters

To confirm that the TCIA dataset did not experience a substantial information loss by the choice of optimized extraction parameters, the features *original_shape_VoxelVolume* and *original_shape_sphericity* were compared after extraction with the default and optimized parameters and did not show any significant differences.

#### 5.1.2. Principal Component Analysis

To further analyze these differences and gain information about the distribution of mutation status within the datasets, dimensionality reduction techniques were applied to gain insights into the data on a global level. The literature presents conflicting findings regarding the visible distinction between wildtypes and KRAS mutant carriers. Pinheiro et al. [17] found no clear distinction between wildtypes and KRAS mutant carriers using PCA and t-SNE on their subset of the TCIA dataset. Oppositely, Prencipe et al. [18] focused on adenocarcinoma and identified a comparably better visible distinction between wildtype and mutant carriers. They used the most meaningful features according to the AUC metric and visualized these differences using t-SNE and Uniform Manifold Approximation and Projection (UMAP).

In this study, both datasets were merged, scaled, and their dimensions reduced following the methodology of Pinheiro et al. [17], given in Figure 4. Aligning with their findings, no clear distinction between wildtypes and KRAS mutant carriers was evident. Instead, two distinct clusters formed, which could be related to nodules and masses in the internal dataset, while the majority of nodules overlapped with the TCIA data points. To gain insights into these clusters and potentially make the ML pipeline more robust, two datasets according to the clusters and a harmonized dataset were created. However, neither of the approaches aided the classification task. The majority of TCIA data points further consisted of lesions with smaller volumes, as apparent in the feature *original_shape_VoxelVolume* (Figure 3), which speaks in favour of the assumption that the TCIA dataset mainly included nodules. As these clusters were still present after dropping the 14 shape features, one can hypothesize that nodules and masses have different radiomic signatures. As many algorithms rely on distance-based methods, such added complexity in the vector space complicates classification tasks. This underscores the need for thoughtful and potentially adaptive dataset creation informed by research outcomes.

Merging radiomic datasets of the same condition might be a more sophisticated task than expected, and should include a thorough analysis of both datasets. As portrayed, increased dataset complexity may arise due to disease characteristics or clinical factors, which may not be directly associated to the classification target. Regarding topics such as federated learning [44], where large parts of the training data remain unknown, such data variability can pose problems and needs to be further investigated.

### 5.2. KRAS Mutation Classification

In our study, several combinations of models, features, and datasets have been applied, aiming to robustly classify KRAS mutation in NSCLC. As the classification problem deals with unbalanced data, the main metric for evaluation was the F1 score, since it balances prediction precision and sensitivity. The best results were observed for the models trained on the balanced dataset, with a mean F1 of 0.68±0.02 (0.68±0.04, respectively) during training and a F1 score of 0.67 during testing, using an ensemble of five models. However, the results are not satisfactory for clinical practice, especially regarding the specificity being effectively zero during training and testing. This portrays one of the downsides of the F1 score, as a high recall (achieved through correctly classifying all KRAS mutated cases) leads to a misleadingly high F1 score. It is therefore crucial to asses model performance across a broad range of metrics.

While slight differences in the metrics can be observed regarding the used dataset in Table 2, the models trained with SMOTE generally showed comparable results during training. These models balanced sensitivity and specificity in their training results. Conversely, the model trained on the balanced dataset maximized F1 by being biased towards one class, which was expected to be mitigated through class balancing without the need for SMOTE. During training and testing, the models trained on the dataset of Moreno et al. stood out by portraying higher scores in their metrics, hinting that their radiomic features expressed the KRAS mutation more adequately leading to a more balanced sensitivity and specificity. Albeit promising results in the work of Moreno et al. [15], ensembles of classifier models did not improve the metrics compared to the single classifier models.

#### 5.2.1. Transferability

In terms of transferability, models’ predictions on the other dataset were not acceptable either, with F1 scores of 0.21 when training on the TCIA data and 0.41 and 0.39 when training on internal data (unbalanced and balanced, respectively). Also, the comparatively high accuracy (0.60) achieved by the XGBoost model trained on the TCIA dataset and tested on the internal dataset can be traced back to the model overclassifying wildtypes incorrectly in an unbalanced dataset. This was reflected in the low sensitivity (0.19) but high specificity (0.76) values, though it was trained on a balanced dataset.

Our findings implicate that the main problem tends to be either in the underlying data or the task itself. Given this subset of the TCIA and internal dataset, a distinction between both mutation types could not be observed visually after PCA, clustering and harmonization, depicted in Figure 4, which raises the question whether ML and radiomics are a sensible approach. Additionally, the referred to ML-based studies found in literature working on the TCIA dataset [15,16,17,18,24] used a different data subset and extracted radiomic features on their own, using potentially different dataset-specific parameters. The results of this study align with Pinheiro et al. [17], who also reported that an acceptable model for KRAS classification could not be obtained.

#### 5.2.2. Related Work

Le et al. [16] trained a model on 143 subjects and tested it on 18 subjects, resulting in a small test group in comparison to the amount of training samples. Three KRAS mutant cases were contained in the test group, from which one was correctly classified. Despite promising results based on the accuracy and AUC metric (0.86 and 0.81, respectively), it remains unclear whether the model generalizes well on a different data split due to the low number of existing and correctly classified mutant cases. Furthermore, the best-performing radiomic ensemble classifier by Moreno et al. [15] demonstrated a discrepancy between sensitivity (0.20) and specificity (0.89), highlighting the challenges in robustly differentiating between the classes. The performance reported by Moreno et al. [15] could not be reached for a variety of possible reasons, such as the data split, the larger test fraction, differing ensemble type, or differences in the training pipeline. Prencipe et al. [18] utilized an approach similar to this study by using different datasets focusing on adenocarcinoma patients only. As they demonstrate promising results on a dataset of 110 patients and did not use the SMOTE algorithm, the further filtering of the underlying data by the NSCLC subtype might be a sensible approach, especially since Pasini et al. [45] reported different radiomic signatures for NSCLC phenotypes.

#### 5.2.3. Data Augmentation

Besides the complexity of capturing KRAS mutations, the question arises whether the SMOTE algorithm adds value within the model training. The generation of synthetic subjects that adequately represent the mutant class seems to be overly complex, given that no differentiation based on the mutation status could be observed on a global level. While the SMOTE algorithm aims to decrease the bias of the majority class by generating samples of the minority class in the training process, it did not enhance the capability of distinguishing between mutant and wildtype lesions in any of the models.

### 5.3. Future Work

Both underlying datasets do not contain information about KRAS submutations [46], which might account for different disease expression in terms of their radiomic features. Submutation distribution could also be a further reason why the datasets differ on a global level and complicate classification tasks. In future work, more finely grained datasets could be generated by including or filtering NSCLC subtypes, as Prencipe et al. [18] carried out, and assessing submutations or other factors potentially complicating the classification task. We found that the consideration of the two data sources through the ComBat data harmonization made the datasets more comparable according to the PCA and t-SNE visualization, but did not improve the model’s capability to differentiate between the two classes. Insights gained from more finely grained datasets might also provide new criteria for data harmonization.

In theory, synthetic subjects could also be generated either on image level or on feature level by means of generative adversarial networks (GANs) [47]. We do not consider this within feasible reach (yet). First, the publicly available training data is very limited in the number of cases, which complicates the generation of images of comparable resolution and quality. Second, the generation of synthetic samples requires a thorough understanding of all covariates, which might have an impact on disease expression in the image. Alternatively, we perceive the possible advantages of federated learning in multi-institutional collaborations, as the cohorts might be composed by matching selection criteria (e.g., treatment status, mutation subtype, scan protocol) and lesion segmentation could be executed in a standardized manner[44]. The similarities of multi-institutional datasets could be compared globally by means of unsupervised distance metrics using either dataset averages or medians or on a feature-level basis. Furthermore, future work could focus on alternative data augmentation techniques such as SMOTE extensions, like k-Means SMOTE [48].

Generally speaking, the presented ML pipeline could be deployed for other classification tasks (e.g., classification of EGFR or ALK mutation instead of KRAS, or tumor phenotyping as originally envisioned for radiomics [49]), and besides radiomic features, any tabular dataset could be used or added. Since the computational costs are low, as no GPU is required to train or test the models, application in a clinical environment appears feasible.

Even though the trained classifiers did not turn out to be suitable for clinical application, we promote further research on the image-based classification of KRAS mutation in NSCLC. While a histologic examination of biopsies is currently the gold standard, this method comes with an uncertainty due to possible sampling errors, albeit due to the presence of multiple lesions or lesion heterogeneity. An image-based quantitative biomarker could therefore be a valuable addition for the determination of risk scores, e.g., combined with lab values, information on smoking history and metastatic burden in multiple regression analyses. In this setting, even incremental improvements of KRAS mutation prediction could positively impact clinical decision making.

### 5.4. Limitations

Despite efforts to mitigate certain factors, there are several underlying limitations to this study that should be investigated in future work. Firstly, the internal dataset is more inhomogeneous than the TCIA dataset. The internal dataset comprises both nodules and masses, which we suspect to have caused two clusters in the t-SNE visualization. Given the differentiation, clustering techniques were applied to create subsets of the data to train expert models, independently from the exact cause of the two observed clusters. As this did not enhance classification performance, future research might group the data points based on alternative criteria.

Also, multiple data harmonization techniques exist to reduce batch effects in the image or feature domain to account for different image acquisition or reconstruction parameters [50]. These have not yet been applied extensively, as we suspect that large differences might already originate from the segmentation process. Different mean HU values and degrees of segmentation fragmentation speak in favor of this assumption. In our study, we considered the two data origins as batches and tried to harmonize the radiomic features accordingly. However, future work could still elaborate on this harmonization technique to further reduce the distance in the vector space between the two datasets, e.g., by considering acquisition or image parameters. The technical possibilities are limited by the comparatively small cohort size, though, as every considered batch or covariate would require a sound number of cases. On the other hand, using a harmonization technique requires access to both datasets, so that the original idea of straightforwardly applying a model trained on public data to a new dataset would not be possible.

Finally, interactions between the mutations present in the datasets, KRAS submutations or NSCLC subtypes, which may influence model performance, were not considered. Enriching and balancing radiomic datasets based on clinical information such as tumor stage, KRAS submutations, or multi-scanner data may yield more promising results in the future.

## 6. Conclusions

This study presented an extensive evaluation of ML algorithms for classifying KRAS mutations in NSCLC using radiomics from two distinct datasets. Although satisfactory classification performance was not achieved, several insights were gained.

First, the importance of data preparation and data validation should not be underestimated for radiomic studies. This was observed in the need for retrospective padding due to differing slice thickness, but also in deviating image orientations or masks not aligning with the image after transforming the images from DICOM into Nifti format using the TCIA data. While there can be several reasons for this, libraries such as PyRadiomics [29] are generally still able to extract radiomic features for potentially faulty image-segmentation pairs.

Although the datasets stem from the same underlying medical condition and capturing method, the two datasets are not alike. This became evident through the statistical analysis of image properties and shape features, as well as visual analysis using dimensionality reduction techniques, which highlighted the global differences between datasets. KRAS mutation tends to have a complex radiomic signature; therefore, differences between wildtypes and mutant carriers could not be observed visually. Also, the tested machine learning models did not achieve robust classification decisions. This became clear in the high variability of the training metrics and generally poor performance on the test sets. Finally, the transferability of models trained on different radiomics datasets did not yield an acceptable prediction outcome, underlining the need for thorough analysis when merging radiomic datasets.

## Figures and Tables

**Table 1:** An overview of the splits into training and testing cases for the four respective dataset variations.

		Training			Testing	
**Dataset**	**Total**	**Wildtype**	**Mutant**	**Total**	**Wildtype**	**Mutant**
TCIA	79	60	19	35	27	8
Moreno et al. [15]	58	44	14	25	19	6
Internal	148	108	40	64	46	18
Internal_balanced_	81	40	41	35	18	17
Cluster 0	112	81	31	49	36	13
Cluster 1	115	86	29	50	38	12
Harmonized	228	169	59	98	72	26

**Table 2:** The three best-performing models during five-fold cross-validation for each dataset, previously sorted by their mean F1 score. The metrics are stated as mean values from the five cross-validation folds, along with the respective standard deviations. For each feature selector, the number of considered features (3, 5, 10, or 15 features) is additionally stated in brackets. Column maxima are highlighted in bold.

Dataset	Classifier	Feature Selector	Mean Accuracy	Mean AUC	Mean F1	Mean Sensitivity	Mean Specificity
TCIA	XGBoost	Spearman (15 F)	0.63 ± 0.15	0.63 ± 0.17	0.42 ± 0.19	0.53 ± 0.23	0.67 ± 0.14
TCIA	XGBoost	MW (15 F)	0.63 ± 0.15	0.63 ± 0.17	0.42 ± 0.19	0.53 ± 0.23	0.67 ± 0.14
TCIA	LR	RFE (3 F)	0.61 ± 0.10	0.60 ± 0.16	0.40 ± 0.22	0.58 ± 0.33	0.62 ± 0.08
Moreno et al.	KNN	MRMR (15 F)	**0.70** ± 0.15	0.66 ± 0.19	0.54 ± 0.11	0.63 ± 0.07	0.71 ± 0.20
Moreno et al.	LR	Pearson (3 F)	0.68 ± 0.12	**0.69** ± 0.15	0.53 ± 0.10	0.73 ± 0.13	0.66 ± 0.14
Moreno et al.	LR	KBest (3 F)	0.68 ± 0.12	**0.69** ± 0.15	0.53 ± 0.10	0.73 ± 0.13	0.66 ± 0.14
Internal	XGBoost	Relief (5 F)	0.59 ± 0.11	0.58 ± 0.08	0.46 ± 0.09	0.62 ± 0.14	0.58 ± 0.14
Internal	KNN	Relief (5 F)	0.59± 0.10	0.54 ± 0.11	0.41 ± 0.13	0.52 ± 0.17	0.61 ± 0.08
Internal	LR	Relief (5 F)	0.58 ± 0.06	0.56 ± 0.06	0.39 ± 0.07	0.50 ± 0.14	0.60 ± 0.10
Internal_balanced_	XGBoost	KBest (10 F)	0.53 ± 0.03	0.49 ± 0.13	**0.68** ± 0.02	0.98 ± 0.05	0.08 ± 0.06
Internal_balanced_	XGBoost	MW (5 F)	0.56 ± 0.07	0.59 ± 0.12	**0.68** ± 0.04	0.95 ± 0.06	0.15 ± 0.15
Internal_balanced_	XGBoost	MRMR (5 F)	0.53 ± 0.04	0.56 ± 0.08	**0.68** ± 0.02	**1.00** ± 0.00	0.05 ± 0.06
Cluster 0	KNN	MRMR (15 F)	0.61 ± 0.10	0.56 ± 0.15	0.43 ± 0.16	0.54 ± 0.23	0.63 ± 0.13
Cluster 0	XGBoost	Pearson (3 F)	0.48 ± 0.07	0.43 ± 0.05	0.42 ± 0.10	0.70 ± 0.22	0.40 ± 0.13
Cluster 0	NN	Pearson (15 F)	0.69 ± 0.07	0.58 ± 0.17	0.41 ± 0.17	0.41 ± 0.21	**0.79** ± 0.10
Cluster 1	KNN	RFE (5 F)	0.60 ± 0.09	0.56 ± 0.16	0.44 ± 0.12	0.63 ± 0.19	0.59 ± 0.07
Cluster 1	KNN	GA (10 F)	0.63 ± 0.07	0.60 ± 0.17	0.42 ± 0.17	0.59 ± 0.30	0.65 ± 0.09
Cluster 1	KNN	Spearman (10 F)	0.54 ± 0.09	0.55 ± 0.13	0.41 ± 0.05	0.63 ± 0.11	0.51 ± 0.13
Harmonized	LR	RFE (3 F)	0.45 ± 0.11	0.46 ± 0.10	0.37 ± 0.06	0.61 ± 0.07	0.39 ± 0.15
Harmonized	NN	GA (15 F)	0.67 ± 0.07	0.58 ± 0.07	0.35 ± 0.08	0.36 ± 0.10	0.78 ± 0.12
Harmonized	XGBoost	KBest (15 F)	0.58 ± 0.07	0.52 ± 0.07	0.34 ± 0.07	0.44 ± 0.14	0.63 ± 0.10

**Table 3:** Model performance on the test data. The best-performing model was tested on the TCIA and internal dataset if possible, before applying the ensemble on the test set. As Moreno et al. [15] did not use PyRadiomics, the model could not be tested on the internal dataset. Column maxima are highlighted in bold.

Trained on	Tested on	Classifier	Feat. Selector	Accuracy	AUC	F1	Sensitivity	Specificity
TCIA	TCIA	XGBoost	Spearman (15 F)	0.54	**0.62**	0.33	0.50	0.56
TCIA	Internal	XGBoost	Spearman (15 F)	0.60	0.47	0.21	0.19	0.76
TCIA	TCIA	Ensemble of 3	Mixed	0.54	0.53	0.33	0.50	0.56
TCIA	TCIA	Ensemble of 5	Mixed	0.54	0.53	0.33	0.50	0.56
TCIA	TCIA	Ensemble of 10	Mixed	0.60	0.56	0.36	0.50	0.63
Moreno et al.	Moreno et al.	KNN	MRMR (15 F)	**0.68**	0.56	0.50	0.67	0.68
Moreno et al.	Moreno et al.	Ensemble of 3	Mixed	0.48	0.54	0.38	0.67	0.42
Moreno et al.	Moreno et al.	Ensemble of 5	Mixed	0.52	0.51	0.33	0.50	0.53
Moreno et al.	Moreno et al.	Ensemble of 10	Mixed	0.56	0.54	0.35	0.50	0.58
Internal	Internal	XGBoost	Relief (5 F)	0.58	0.56	0.49	0.72	0.52
Internal	TCIA	XGBoost	Relief (5 F)	0.46	0.55	0.41	0.78	0.37
Internal	Internal	Ensemble of 3	Mixed	0.50	0.50	0.36	0.50	0.50
Internal	Internal	Ensemble of 5	Mixed	0.56	0.58	0.44	0.61	0.54
Internal	Internal	Ensemble of 10	Mixed	0.52	0.48	0.31	0.39	0.57
Internal_balanced_	Internal_balanced_	XGBoost	KBest (10 F)	0.49	0.33 *	0.65	**1.00**	0.00
Internal_balanced_	TCIA	XGBoost	KBest (10 F)	0.25	0.44	0.39	**1.00**	0.01
Internal_balanced_	Internal_balanced_	Ensemble of 3	Mixed	0.49	0.50 *	0.65	**1.00**	0.00
Internal_balanced_	Internal_balanced_	Ensemble of 5	Mixed	0.51	0.53	**0.67**	**1.00**	0.06
Internal_balanced_	Internal_balanced_	Ensemble of 10	Mixed	0.49	0.50 *	0.65	**1.00**	0.00
Cluster 0	Cluster 0	KNN	MRMR (15 F)	0.49	0.46	0.32	0.46	0.50
Cluster 0	Cluster 0	Ensemble of 3	Mixed	0.45	0.38	0.18	0.23	0.53
Cluster 0	Cluster 0	Ensemble of 5	Mixed	0.51	0.40	0.14	0.15	0.64
Cluster 0	Cluster 0	Ensemble of 10	Mixed	0.57	0.44	0.16	0.15	0.72
Cluster 1	Cluster 1	KNN	RFE (5 F)	0.48	0.38	0.24	0.33	0.53
Cluster 1	Cluster 1	Ensemble of 3	Mixed	0.60	0.51	0.29	0.33	0.68
Cluster 1	Cluster 1	Ensemble of 5	Mixed	0.58	0.47	0.22	0.25	0.68
Cluster 1	Cluster 1	Ensemble of 10	Mixed	0.58	0.47	0.22	0.25	0.68
Harmonized	Harmonized	LR	RFE (3 F)	0.62	0.51	0.33	0.35	0.72
Harmonized	Harmonized	Ensemble of 3	Mixed	0.65	0.52	0.26	0.23	**0.81**
Harmonized	Harmonized	Ensemble of 5	Mixed	0.60	0.51	0.29	0.31	0.71
Harmonized	Harmonized	Ensemble of 10	Mixed	0.59	0.45	0.17	0.15	0.75

* The AUC differs for these models, since the calculation was not based on predicted probabilities in the hard voting ensemble.

## Data Availability

The radiomics data supporting the conclusions of this article will be made available by the authors on request, while the underlying imaging data can be inspected on-premise. The public dataset used in this study is openly available in The Cancer Imaging Archive at https://www.cancerimagingarchive.net/collection/nsclc-radiogenomics/, accessed on 9 February 2023.

## Abbreviations

AI	Artificial Intelligence
ALK	Anaplastic lymphoma kinase
AUC	Area Under the Curve
CNNs	Convolutional Neural Networks
EGFR	Epidermal Growth Factor Receptor
FGFR	Fibroblast Growth Factor Receptor
GA	Genetic Algorithm
GAN	Generative Adversarial Network
HU	Hounsfield Unit
IBSI	Image Biomarker Standardisation Initiative
KNN	K-Nearest Neighbors
KRAS	Kirsten Rat Sarcoma (viral oncogene homolog)
LR	Logistic Regression
ML	Machine Learning
MRMR	Maximum Redundancy Maximum Relevance
MW	Mann–Whitney U
NN	Neural Networks
NSCLC	Non-small-cell lung cancer
PACS	Picture Archiving and Communication System
PCA	Principal Component Analysis
RF	Random Forest
RFE	Recursive Feature Elimination
SCLC	Small-cell lung cancer
SMOTE	Synthetic Minority Over-sampling Technique
t-SNE	t-Distributed Stochastic Neighbor Embedding
TCIA	The Cancer Imaging Archive
UMAP	Uniform Manifold Approximation and Projection
XGBoost	Extreme Gradient Boosting
